# Bilateral Patellar Tendon Rupture in a 64‐Year‐Old Man: Possible Interaction of Inhaled Corticosteroids, Age and Mechanical Stress

**DOI:** 10.1155/cro/9959487

**Published:** 2026-06-02

**Authors:** Mohamed Jalaleddin, Pavun Basra, Christopher Jordan, Benjamin H. L. Harris, Louis J. Koizia

**Affiliations:** ^1^ Imperial College Healthcare NHS Trust, St. Mary′s Hospital, London, UK, nhs.uk; ^2^ Cutrale Perioperative and Ageing Group, Department of Bioengineering, Imperial College London, London, UK, imperial.ac.uk

**Keywords:** bilateral, patellar tendon, spontaneous rupture

## Abstract

Bilateral rupture of the patellar tendon is a rare occurrence, most often associated with systemic disease or exposure to systemic corticosteroids or fluoroquinolones. We report the case of a 64‐year‐old man with bronchiectasis managed with long‐term inhaled corticosteroids who sustained bilateral patellar tendon rupture following a low‐energy deceleration while crossing the road. He underwent bilateral patellar tendon repair with medial and lateral retinacular reinforcement and was managed postoperatively with a structured rehabilitation programme. At 28‐week follow‐up, he remained independently ambulant with no evidence of tendon re‐rupture. The patient had several recognised contributors to tendon vulnerability, including advanced age and prolonged inhaled corticosteroid exposure. Although inhaled corticosteroids confer lower systemic bioavailability than oral preparations, long‐term use may contribute to tendon vulnerability, particularly when combined with additional mechanical or degenerative factors; however, causality cannot be confirmed. No histological analysis, preinjury imaging, or biochemical tendon assessment were available to characterise baseline tendon integrity. This case highlights the importance of enquiring about chronic inhaled corticosteroid use and considering the additive interaction of multiple risk factors when evaluating spontaneous tendon rupture.

## 1. Background

Patellar tendon rupture is an uncommon injury, with bilateral cases being exceedingly rare, especially in the absence of trauma or systemic disease. Most reported cases occur in individuals with underlying risk factors such as diabetes, chronic kidney disease, rheumatoid arthritis, or other systemic conditions affecting tendon integrity [1]. In addition to systemic disease, pharmacological exposures, most notably systemic corticosteroids, are well‐recognised contributors to tendon rupture. Fluoroquinolone antibiotics have also been implicated in tendon rupture due to their impact on collagen integrity [2]. Although most cases occur in patients with recognised risk factors, bilateral rupture remains uncommon and may occur in the presence of multiple interacting vulnerabilities [3].

This case describes bilateral low‐energy patellar tendon rupture in a 64‐year‐old man with recognised risk factors for tendon vulnerability, including advanced age, long‐term inhaled corticosteroid exposure and a history of long‐distance running. Unlike systemic corticosteroid therapy, inhaled corticosteroids have lower systemic bioavailability; however, prolonged exposure may still contribute to tendon vulnerability, particularly in the presence of additional mechanical or degenerative factors. We compare this presentation with previously reported cases and discuss potential mechanisms contributing to tendon failure in the context of cumulative corticosteroid exposure and age‐related tendon changes.

## 2. Case Presentation

### 2.1. Patient Background

A 64‐year‐old man with bronchiectasis (diagnosed in 2016 and managed with long‐term inhaled corticosteroids) and chronic cervical neck pain presented with acute bilateral knee pain. His regular medications included beclometasone/formoterol inhaler (100 mcg/dose, two puffs twice daily), gabapentin 100 mg three times daily and codeine 30 mg twice daily. The patient had used inhaled beclometasone at a total daily dose of 400 mcg for approximately 7 years. He had no history of oral corticosteroid therapy, fluoroquinolone use, systemic inflammatory disease, or metabolic disorders. His body mass index was 24 kg/m^2^, and he only smoked socially in his early twenties. Baseline blood tests were normal including full blood count, renal function, calcium and phosphate.

### 2.2. Mechanism of Injury

The patient, a previously active individual and former long‐distance runner, suddenly decelerated while crossing the road to avoid an approaching vehicle. He immediately experienced bilateral knee pain and collapse, with an inability to bear weight. He denied any preceding knee pain, instability, constitutional symptoms, or recent infection.

### 2.3. Examination and Imaging

On examination, both knees demonstrated:

• Visible effusions
• Apparent superior displacement of both patellae (suggestive of patella alta)
• Inability to perform active knee extension
• Tenderness over both patellar tendons

**Figure 1:**
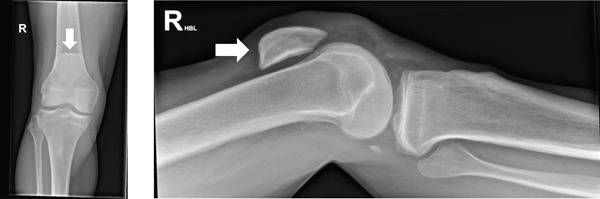
Right knee radiograph demonstrating superior displacement of the patella (arrow), consistent with patella alta and disruption of the extensor mechanism, in keeping with patellar tendon rupture.

**Figure 2:**
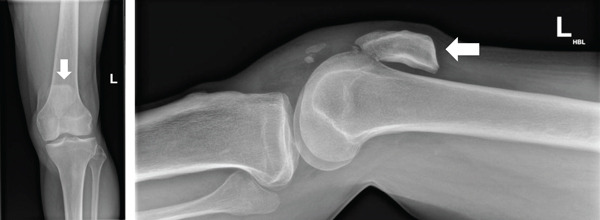
Left knee radiograph demonstrating superior displacement of the patella (arrow), consistent with patella alta and disruption of the extensor mechanism, in keeping with patellar tendon rupture.

Plain radiographs demonstrated bilateral patellar tendon rupture with superior displacement of both patellae (Figures 1 and 2, apparent superior displacement of both patellae shown with arrows). Formal measurement of patellar height indices such as the Insall–Salvati ratio was not undertaken, as the radiographs did not meet standard positioning criteria (approximately 30° knee flexion) required for reliable assessment. Patellar position was therefore assessed qualitatively.

### 2.4. Surgical Findings

The patient underwent bilateral patellar tendon repair with medial and lateral retinacular repair. Direct end‐to‐end repair was performed and augmented using nonabsorbable suture reinforcement. Intraoperatively, there was no macroscopic evidence of tendinosis, degeneration, or intra‐articular pathology.

### 2.5. Postoperative Care

Postoperatively, the patient was managed on a structured, phased rehabilitation programme led by the surgical and physiotherapy teams:

• Weeks 0–2: Hinged knee braces locked in extension; partial weight‐bearing as tolerated.
• Weeks 2–6: Flexion increased in 30° increments every 2 weeks; supervised quadriceps isometric strengthening initiated.
• By Week 6: The patient achieved 0°–60° bilateral knee flexion with intact active extension and improving quadriceps activation.
• Weeks 6–12: Progressive functional rehabilitation and strengthening.

At early follow‐up, he was independently ambulant with no extensor lag. Formal functional scores were not obtained at this stage. At 28‐week follow‐up, the patient remained independently ambulant with no evidence of tendon re‐rupture.

### 2.6. Additional Investigations

• Rheumatoid factor < 20 (negative)
• Cyclic citrullinated peptide antibodies 1.2 (negative)

### 2.7. Diagnostic Limitations

This case has several limitations that should be considered when interpreting the findings. While structural and biochemical characterisation was incomplete, these constraints reflect real‐world clinical practice, where comprehensive baseline data are often unavailable. As such, the findings should be interpreted as hypothesis‐generating, particularly in relation to the potential role of long‐term inhaled corticosteroid exposure in tendon pathology.

## 3. Discussion

Spontaneous rupture of the patellar tendon is an uncommon but clinically significant injury, typically associated with systemic disease, chronic degenerative changes, or pharmacological exposure. Unlike traumatic ruptures, which usually result from high‐energy injuries, spontaneous ruptures arise from pre‐existing tendon pathology that predisposes the tissue to failure [4]. Recognition of predisposing factors and timely intervention are important to optimise outcomes.

Pharmacological agents are also established risk factors. Chronic systemic corticosteroid therapy suppresses fibroblast proliferation and impairs collagen synthesis, thereby reducing tendon tensile strength [5]. Fluoroquinolone antibiotics such as ciprofloxacin and levofloxacin have likewise been linked to spontaneous tendon rupture through matrix metalloproteinase activation and collagen degradation pathways [2]. Immunosuppressive medications used in autoimmune conditions may further impair tendon health [6].

A variety of systemic conditions contribute to the pathogenesis of spontaneous patellar tendon rupture [6]. Chronic kidney disease and end‐stage renal disease are well‐recognised contributors due to disruptions in calcium–phosphate homeostasis and secondary hyperparathyroidism, which promote collagen degradation [1]. Similarly, diabetes mellitus, rheumatoid arthritis, systemic lupus erythematosus and primary hyperparathyroidism have been implicated in progressive tendinopathy and impaired collagen synthesis [7], whereas gout may weaken tendons through urate crystal deposition and local inflammation [8].

Mechanical stress also plays a key role in tendon vulnerability. Chronic repetitive loading and microtrauma contribute to collagen disorganisation and structural failure [4]. Patellar tendinosis (jumper′s knee) is a recognised precursor, particularly in individuals engaged in repetitive high‐impact activities [2, 9], and repeated corticosteroid injections have been shown to weaken tendon structure [10]. In this patient, a history of long‐distance running may have resulted in cumulative microtrauma, whereas age‐related degenerative changes likely reduced tensile resilience. These interacting factors may have lowered the threshold for rupture during sudden deceleration.

Clinically, spontaneous patellar tendon rupture presents with acute knee pain, swelling and inability to actively extend the knee, reflecting the loss of extensor mechanism integrity [1]. Imaging supports diagnosis: plain radiographs may demonstrate features suggestive of patella alta, which can be quantified using the Insall–Salvati ratio [11]. Ultrasound may aid in rapid assessment, whereas MRI remains the gold standard for detailed evaluation of tendon integrity and associated pathology [12]. Complete ruptures require prompt surgical repair, typically via primary reattachment using transosseous sutures or suture anchors [12, 13], followed by structured rehabilitation [14]. Partial ruptures may be managed nonoperatively in selected cases [15].

Importantly, bilateral patellar tendon rupture is not exclusively confined to patients with recognised risk factors. Cases have been reported in otherwise healthy individuals, and approximately one‐third of patients in published case series have no identifiable predisposing condition [16, 17]. This suggests that tendon rupture may occur in the presence of subclinical or unmeasured factors, including early degenerative changes, genetic predisposition, or cumulative microtrauma. Future research using genome‐wide association studies and other large‐scale data‐driven approaches [18–20], as demonstrated in other fields, may help to elucidate underlying susceptibility.

This case contributes to the existing literature by demonstrating how multiple modest risk factors, including advanced age, cumulative mechanical loading and prolonged inhaled corticosteroid exposure, may act synergistically to reduce tendon resilience, even in the absence of systemic disease or significant trauma. This is clinically important, as inhaled corticosteroids are often perceived as low risk and their potential contribution to tendon pathology may therefore be underrecognised in routine assessment.

Notably, the absence of macroscopic degeneration at surgery may argue against advanced structural tendinopathy and weakens a direct causal inference regarding corticosteroid exposure. This raises the possibility that tendon failure occurred in the presence of subclinical or microscopic changes, or through the interaction of multiple contributing factors lowering the threshold for rupture.

This case has diagnostic limitations. Intraoperative inspection cannot exclude microscopic tendinopathy, and histopathological analysis was not performed. MRI was not obtained prior to urgent repair [12]. Although patellar height can be assessed using indices such as the Insall–Salvati ratio [11], the available radiographs did not meet standard positioning criteria (approximately 30° of knee flexion) required for reproducible measurement. As such, formal calculation was not performed, and patella alta could not be objectively confirmed. Laboratory evaluation was not exhaustive; vitamin D and parathyroid hormone levels were not assessed [21], and genetic factors influencing collagen integrity were not investigated [22]. These limitations restrict definitive characterisation of baseline tendon health. Follow‐up was limited to 28 weeks, and although early outcomes were favourable, including absence of extensor lag and return to independent ambulation, longer term functional outcomes remain unknown.

Overall, spontaneous rupture of the patellar tendon remains rare but clinically important. In this case, recognised risk factors for tendon vulnerability, including advanced age, chronic inhaled corticosteroid exposure and prior mechanical loading, may have contributed to lowering the threshold for bilateral rupture.

## 4. Conclusion

This case report describes bilateral low‐energy patellar tendon rupture in a 64‐year‐old man with recognised contributors to tendon vulnerability, including advanced age, prolonged inhaled corticosteroid exposure and prior mechanical loading. Although bilateral rupture remains rare, the cumulative interaction of pharmacological, degenerative and biomechanical factors may lower the threshold for tendon failure. Clinicians should routinely enquire about long‐term inhaled corticosteroid use when evaluating spontaneous tendon rupture and consider the potential additive effect of multiple risk factors rather than focusing on a single precipitant. However, longer term outcomes and patient‐reported measures are required to better understand functional recovery.

## Author Contributions

The authors state equal contributions in the preparation of this manuscript.

## Funding

No funding was received for this manuscript.

## Ethics Statement

Ethical approval was not required for a single‐patient case report. Written informed consent for publication of clinical details and images was obtained from the patient.

## Conflicts of Interest

The authors declare no conflicts of interest.

## Data Availability

Data sharing is not applicable to this article as no datasets were generated or analysed during the current study.
